# COVID-19 Organ Injury Pathology and D-Dimer Expression Patterns: A Retrospective Analysis

**DOI:** 10.3390/diagnostics15151860

**Published:** 2025-07-24

**Authors:** Raluca Dumache, Camelia Oana Muresan, Sorina Maria Denisa Laitin, Nina Ivanovic, Adina Chisalita, Alexandra Herlo, Adelina Marinescu, Elena Voichita Lazureanu, Talida Georgiana Cut

**Affiliations:** 1 Department of Forensic Medicine, Bioethics, Medical Ethics and Medical Law, “Victor Babes” University of Medicine and Pharmacy, Eftimie Murgu Square 2, 300041 Timisoara, Romania; raluca.dumache@umft.ro; 2 Center for Ethics in Human Genetic Identifications, “Victor Babes” University of Medicine and Pharmacy, Eftimie Murgu Square 2, 300041 Timisoara, Romania; nina.ivanovic@umft.ro (N.I.); adina.chisalita@umft.ro (A.C.); talida.cut@umft.ro (T.G.C.); 3 Department of Epidemiology, “Victor Babes” University of Medicine and Pharmacy, Eftimie Murgu Square 2, 300041 Timisoara, Romania; 4 Doctoral School, “Victor Babes” University of Medicine and Pharmacy, Eftimie Murgu Square 2, 300041 Timisoara, Romania; 5 Department of Infectious Diseases, “Victor Babes” University of Medicine and Pharmacy, Eftimie Murgu Square 2, 300041 Timisoara, Romania; alexandra.mocanu@umft.ro (A.H.); adelina.marinescu@umft.ro (A.M.); lazureanu.voichita@umft.ro (E.V.L.)

**Keywords:** COVID-19, SARS-CoV-2, diagnosis, biomarkers, pathology, D-dimer, immunity and inflammation mediators, organ injury, coagulopathy

## Abstract

**Background and Objectives:** Coronavirus Disease 2019 (COVID-19) may cause extensive multi-organ pathology, particularly in the lungs, heart, kidneys, and liver. While hypercoagulability—often signaled by elevated D-dimer—has been thoroughly investigated, the concurrent pathological findings across organs and their interrelation with distinct D-dimer levels remain incompletely characterized. This study aimed to evaluate the pathological changes observed in autopsied or deceased COVID-19 patients, focusing on the prevalence of organ-specific lesions, and to perform subgroup analyses based on three D-dimer categories. **Methods:** We conducted a retrospective review of 69 COVID-19 patients from a Romanian-language dataset, translating all clinical and pathological descriptions into English. Pathological findings (pulmonary microthrombi, bronchopneumonia, myocardial fibrosis, hepatic steatosis, and renal tubular necrosis) were cataloged. Patients were grouped into three categories by admission D-dimer: <500 ng/mL, 500–2000 ng/mL, and ≥2000 ng/mL. Laboratory parameters (C-reactive protein, fibrinogen, and erythrocyte sedimentation rate) and clinical outcomes (intensive care unit [ICU] admission, mechanical ventilation, and mortality) were also recorded. Intergroup comparisons were performed with chi-square tests for categorical data and one-way ANOVA or the Kruskal–Wallis test for continuous data. **Results:** Marked organ pathology was significantly more frequent in the highest D-dimer group (≥2000 ng/mL). Pulmonary microthrombi and bronchopneumonia increased stepwise across ascending D-dimer strata (*p* < 0.05). Myocardial and renal lesions similarly showed higher prevalence in patients with elevated D-dimer. Correlation analysis revealed that severe lung and heart pathologies were strongly associated with high inflammatory markers and a greater risk of ICU admission and mortality. **Conclusions:** Our findings underscore that COVID-19-related organ damage is magnified in patients with significantly elevated D-dimer. By integrating pathology reports with clinical and laboratory data, we highlight the prognostic role of hypercoagulability and systemic inflammation in the pathogenesis of multi-organ complications. Stratifying patients by D-dimer may inform more tailored management strategies, particularly in those at highest risk of severe pathology and adverse clinical outcomes.

## 1. Introduction

Coronavirus Disease 2019 (COVID-19), caused by the novel coronavirus SARS-CoV-2, continues to exert a formidable global health impact [1]. Although vaccination programs and improved therapies have mitigated mortality, a substantial number of patients progress to severe disease, especially those with comorbidities and dysregulated immune responses [2,3]. Early in the pandemic, scholars observed coagulopathy and thrombosis as major contributors to morbidity and mortality [4]. However, it became evident that COVID-19 is more than just a respiratory infection—it involves a complex pathophysiological interplay of hyperinflammation, endothelial dysfunction, and multi-organ injury [5].

A central aspect of COVID-19 coagulopathy is the elevated D-dimer, reflecting ongoing fibrin formation and degradation [6,7]. Numerous investigations identify high D-dimer as a robust predictor of adverse outcomes, including respiratory failure and death [6,8]. While the mechanistic underpinnings of D-dimer elevation in COVID-19 are multifactorial—ranging from microvascular endothelial injury to immunothrombosis—there is still scope for refined subgroup analysis. Many of the existing data focus primarily on vascular thrombotic events, whereas fewer studies extend their attention to the pathological correlates in the lungs, heart, kidneys, and liver that accompany these high D-dimer states [4,9].

Autopsy and surgical pathology studies highlight that severe COVID-19 frequently features pulmonary microthrombi, diffuse alveolar damage, and inflammatory cell infiltrates [9,10]. Cardiac specimens often demonstrate myocardial fibrosis or even microinfarcts, while kidneys can exhibit acute tubular necrosis or microangiopathy [9,11]. Hepatic findings vary from mild fatty changes to severe congestion or necrosis [12]. These pathologies likely stem from a combination of direct viral invasion, cytokine storm, and hypercoagulability [13,14]. Unraveling how such findings are distributed across different degrees of D-dimer elevation could deepen the clinical understanding of disease trajectories.

Hyperinflammation and hypercoagulability in COVID-19 appear to be tightly interwoven, creating a positive feedback loop capable of damaging multiple organ systems [15,16]. Mechanistically, SARS-CoV-2 elicits a systemic inflammatory cascade—often termed the “cytokine storm”—and triggers endothelial cell activation [17]. This endothelial dysfunction can expose the subendothelial collagen layer, amplifying the coagulation cascade [18]. Concurrently, sustained inflammation can worsen local tissue injury, favoring microthrombi formation and further organ compromise [19,20]. Detailed autopsy-driven evidence clarifies the morphological features (e.g., fibrin deposits, necrosis) that coincide with biomarker surges, such as D-dimer and CRP.

COVID-19-associated coagulopathy, typified by sharp rises in D-dimer, has been repeatedly linked to thrombotic complications; nevertheless, the extent to which progressively higher D-dimer strata map onto concrete, organ-specific histopathologies remains underexplored. We therefore aimed to test the hypothesis that increasing admission D-dimer levels (<500 ng/mL, 500–2000 ng/mL, and ≥2000 ng/mL) are associated with a stepwise rise in the prevalence and severity of lung, cardiac, renal, and hepatic lesions in hospitalized COVID-19 patients.

## 2. Materials and Methods

### 2.1. Study Design and Setting

This retrospective study took place at a tertiary medical center in Timisoara, Romania, at the Victor Babes University of Medicine and Pharmacy. We analyzed 69 patients with confirmed COVID-19 (via RT-PCR) between 2020 and 2023. Data were abstracted retrospectively from a mandatory, prospectively maintained electronic COVID-19 registry used by all wards of the hospital. Clinical data, laboratory values, and pathology reports (autopsies or surgical specimens) were originally documented in the database and paper records. For uniformity, all terms related to organ findings were translated into their English equivalents. The hospital primarily served patients from both urban and rural regions, admitting severe COVID-19 cases. All pathological reports were reviewed by two independent pathologists. Ethical approval was obtained from the institutional review board (no 5058/28.05.2021), and consent requirements were waived due to the study’s retrospective and de-identified nature.

### 2.2. Inclusion Criteria and Data Extraction

Patients were eligible if they had (1) a confirmed SARS-CoV-2 infection; (2) documented D-dimer at hospital admission; (3) available data on key inflammatory markers (C-reactive protein, fibrinogen, and ESR); and (4) pathologic findings describing at least one organ system (lung, heart, kidney, or liver) either ante-mortem or post-mortem. We excluded cases with incomplete lab data or ambiguous cause of death unrelated to COVID-19. We extracted demographic variables (age, sex), comorbid conditions (hypertension, diabetes mellitus, chronic kidney disease, obesity [BMI ≥ 30 kg/m^2^], chronic obstructive pulmonary disease, coronary-artery disease, or active malignancy), vital parameters, and final outcomes (ICU admission, mechanical ventilation, and in-hospital mortality). For pathology, we recorded the presence or absence of specific lesions: pulmonary microthrombi, bronchopneumonia, diffuse alveolar damage, myocardial fibrosis, myocardial infarction, renal tubular necrosis, and hepatic steatosis. Additional observations (e.g., hemorrhagic necrosis and vascular congestion) were also noted. All Romanian-language clinical and pathology reports were independently translated into English by two researchers participating in the study.

### 2.3. D-Dimer Subgroup Classification

Patients were categorized into three D-dimer strata based on admission levels (ng/mL): Group 1: <500 (“normal/low”); Group 2: 500–2000 (“moderately elevated”); and Group 3: ≥2000 (“markedly elevated”). These thresholds coincide with those repeatedly linked to sharp inflection points in mortality, and are endorsed by several consensus guidelines [7,14]. Other laboratory values included CRP (mg/L), ESR (mm/h), and fibrinogen (mg/dL). CRP ≥ 50 mg/L, ESR > 40 mm/h, and fibrinogen ≥ 400 mg/dL were considered “elevated” in a broad clinical sense [20].

We also noted whether patients underwent anticoagulation (prophylactic or therapeutic dose) during hospitalization, as this may influence D-dimer dynamics and overall outcomes. However, due to incomplete records on specific anticoagulant regimens, these data were not included in the comparative statistics, limiting our ability to control for treatment heterogeneity in the final analysis.

D-dimer was measured via a latex-enhanced immunoturbidimetric assay (ACL TOP 700, Instrumentation Laboratory, Bedford, MA, USA) and reported in fibrinogen equivalent units (FEU; reference < 500 ng/mL). C-reactive protein concentrations were quantified via high-sensitivity immunonephelometry on a BN II System analyser (Siemens Healthineers, Erlangen, Germany), with a reference range of <5 mg/L. Plasma fibrinogen was measured via the Clauss clot-based assay on the same ACL TOP 700 platform as D-dimer, with 200–400 mg/dL taken as the reference interval. The erythrocyte sedimentation rate was determined within two hours of venepuncture using the fully automated Westergren-based Alifax Roller 20 instrument (Alifax S.p.A., Padua, Italy); values ≤ 20 mm/h were considered normal. All laboratory parameters cited herein were obtained within six hours of hospital admission.

### 2.4. Statistical Analysis

All statistical analyses were performed using SPSS version 27 (IBM Corp., Armonk, NY, USA). Continuous data were tested for normality (Shapiro–Wilk test). For normally distributed variables, results are reported as mean ± standard deviation and compared using one-way ANOVA. Kruskal–Wallis tests were used for non-normally distributed variables, reported as the median (interquartile range). Categorical data (e.g., presence or absence of a given pathology) were compared using chi-square or Fisher’s exact tests, as appropriate. We created correlation matrices (Spearman’s or Pearson’s r) to evaluate associations among lab parameters (D-dimer, CRP, ESR, and fibrinogen) and pathology findings. A multivariable logistic model (enter method) included age, sex, obesity, chronic kidney disease, and D-dimer ≥ 2000 ng/mL; only variables with *p* < 0.10 in univariate screening were retained. Multiple comparisons among the three D-dimer groups included post hoc tests, with *p*-values < 0.05 indicating significance. All *p*-values were two-sided.

## 3. Results

Table 1 provides a broad overview of the 69 patients included in this study, summarizing their demographic backgrounds and key comorbidities without subdividing them by D-dimer level. The average age was around 63 years. Males constitute nearly 60% of the cohort. Hypertension emerged as the most prevalent comorbidity, present in over half of the patients. Around one-fifth of patients had diabetes mellitus, while approximately the same proportion were diagnosed with chronic kidney disease. Twelve of these patients (17.4%) had a history of malignancy. Moreover, pulmonary pathology dominates, with diffuse alveolar damage (43.5%) and bronchopneumonia (40.6%) prevalent. Pulmonary microthrombi appear in 37.7% of cases. Myocardial fibrosis is present in almost half of the cohort, renal tubular necrosis appears in one-third (33.3%) of patients, hepatic steatosis is noted in 27.5%, while recent myocardial infarction is found in 15.9%.

Compared with patients in the lowest D-dimer category (<500 ng/mL), those with intermediate (500–2000 ng/mL) and high (≥2000 ng/mL) D-dimer levels exhibited a progressive increase in the prevalence of most comorbid conditions. While hypertension was similarly common across groups (45.0%, 55.6%, and 54.5%, respectively; *p* = 0.64), diabetes mellitus rose from 10.0% to 22.2% and 31.8% (*p* = 0.14), and obesity increased from 15.0% to 18.5% and 31.8% (*p* = 0.31). Notably, chronic kidney disease showed a statistically significant escalation, affecting 5.0%, 18.5%, and 36.4% of patients in the low, intermediate, and high D-dimer groups, respectively (*p* = 0.01). Although the prevalence of coronary-artery disease and COPD also trended upward with higher D-dimer levels, these differences did not reach significance (*p* = 0.24 and *p* = 0.28). Active malignancy rates remained stable across strata (approximately 15–18%, *p* = 0.91). Overall, the mean number of comorbidities per patient increased significantly from 1.3 ± 0.9 in the lowest category to 1.7 ± 1.0 and 2.2 ± 1.1 in the intermediate and highest categories, respectively (*p* = 0.006), underscoring a greater comorbidity burden in patients with elevated D-dimer levels (Table 2).

Table 3 classifies the 69 patients into three D-dimer strata: <500 ng/mL, 500–2000 ng/mL, and ≥2000 ng/mL. Interestingly, the distribution across groups is relatively balanced, though the “moderately elevated” subset (500–2000 ng/mL) is the largest, capturing nearly 40% of the cohort. Just under one-third present with normal/low D-dimer (<500 ng/mL), while about the same proportion display markedly elevated levels (≥2000 ng/mL).

Table 4 illustrates the incidence of various organ pathologies across three D-dimer groups. The frequency of pulmonary microthrombi increases with higher D-dimer levels, with 20.0% (4 out of 20 participants) in Group 1 (<500), 37.0% (10 out of 27 participants) in Group 2 (500–2000), and 54.5% (12 out of 22 participants) in Group 3 (≥2000), showing a statistically significant difference, with a *p*-value of 0.02. Similarly, the prevalence of bronchopneumonia also shows a significant increase across the groups: 25.0% (5 out of 20) in Group 1, 37.0% (10 out of 27) in Group 2, and 59.1% (13 out of 22) in Group 3, with a *p*-value of 0.04.

Other pathologies, although showing upward trends, did not reach statistical significance. Myocardial fibrosis was observed in 30.0% (6 out of 20) of Group 1, increasing to 48.1% (13 out of 27) in Group 2, and 54.5% (12 out of 22) in Group 3, with a *p*-value of 0.09. Renal tubular necrosis was reported in 20.0% (4 out of 20) of Group 1, 33.3% (9 out of 27) in Group 2, and 45.5% (10 out of 22) in Group 3, with a *p*-value of 0.08. Hepatic steatosis increased from 15.0% (3 out of 20) in Group 1 to 29.6% (8 out of 27) in Group 2, and 36.4% (8 out of 22) in Group 3, showing a *p*-value of 0.13. These findings underscore that an admission D-dimer ≥ 2000 ng/mL is not merely a laboratory anomaly, but a phenotypic marker of diffuse microvascular injury, especially in the lungs, where microthrombi were detected in over half of the patients.

Table 5 evaluates whether inflammatory markers parallel changes in D-dimer. Across the three subgroups, CRP, ESR, and fibrinogen all rise significantly as D-dimer levels escalate (*p* < 0.001, *p* = 0.002, and *p* = 0.001, respectively). The median CRP in Group 1 is 24.5 mg/L, consistent with a moderate inflammatory state, whereas Group 3’s median approaches 92.4 mg/L, indicative of marked systemic inflammation. Similarly, ESR exhibits a near-doubling from Group 1 (median 25 mm/h) to Group 3 (58 mm/h), and mean fibrinogen climbs from 348 mg/dL to 478 mg/dL.

Table 6 confirms that ascending D-dimer levels tie closely to poorer clinical outcomes in COVID-19. ICU admission rates climb markedly from 20% in Group 1 to 54.5% in Group 3 (*p* = 0.01). While mechanical ventilation shares a similar upward trend (10% → 36.4%), the *p*-value is marginally above significance (*p* = 0.06). Mortality showed a dramatic rise from 5% in the low D-dimer group to over one-third (36.4%) in the ≥2000 ng/mL group (*p* = 0.01). Furthermore, the median length of stay (LOS) increases by nearly a week between Groups 1 and 3 (9 vs. 15 days).

The analysis showed higher incidences of all listed conditions in the CKD present group compared to the CKD absent group. Specifically, pulmonary microthrombi were observed in 64.3% (9 out of 14) of patients with CKD, significantly higher than the 30.9% (17 out of 55) seen in those without CKD, with a *p*-value of 0.01. Additionally, bronchopneumonia and myocardial fibrosis were both reported in 71.4% (10 out of 14) of CKD patients, compared to 32.7% (18 out of 55) and 38.2% (21 out of 55), respectively, in non-CKD patients, with *p*-values of 0.003 and 0.02, respectively. Renal tubular necrosis was particularly high in the CKD group at 78.6% (11 out of 14), compared to only 21.8% (12 out of 55) in those without CKD, with a highly significant *p*-value of less than 0.001. Markedly elevated D-dimer levels (≥2000) were also significantly more frequent in CKD patients, 64.3% (9 out of 14) versus 23.6% (13 out of 55), with a *p*-value of 0.002. Finally, mortality was markedly higher in the CKD group, affecting 57.1% (8 out of 14) compared to 9.1% (5 out of 55), with a *p*-value of less than 0.001 (Table 7).

The data indicated higher rates of each condition among patients with diabetes. In the group with diabetes, 66.7% (10 out of 15) had markedly elevated D-dimer levels (≥2000 ng/mL), significantly more than the 22.2% (12 out of 54) observed in those without diabetes, with a *p*-value of 0.001. Pulmonary microthrombi were present in 73.3% (11 out of 15) of patients with diabetes, compared to only 27.8% (15 out of 54) of those without diabetes, with a *p*-value of 0.002. Renal tubular necrosis was reported in 60.0% (9 out of 15) of diabetic patients versus 25.9% (14 out of 54) of non-diabetic patients, with a *p*-value of 0.02. Myocardial fibrosis was noted in 66.7% (10 out of 15) of the diabetes group compared to 38.9% (21 out of 54) in the non-diabetes group, with a *p*-value of 0.06, suggesting a trend towards significance but not reaching the statistical threshold. Furthermore, 73.3% (11 out of 15) of diabetic patients had two or more organ pathologies, significantly higher than 38.9% (21 out of 54) in those without diabetes, with a *p*-value of 0.01. Lastly, in-hospital mortality was notably higher among diabetic patients at 46.7% (7 out of 15), compared to 9.3% (5 out of 54) in those without diabetes, with a *p*-value of less than 0.001 (Table 8).

The cross-tabulation confirms a clear, dose–response relationship between admission D-dimer and multimorbidity burden. Among patients with low D-dimer (<500 ng/mL), the great majority (70%) had no more than one chronic condition, and their mean comorbidity count was 1.3 ± 0.9. In the intermediate stratum (500–2000 ng/mL), this balance inverted: almost three-fifths (59.3%) harbored two or more comorbidities, raising the average to 1.7 ± 1.0. The gradient steepened further in the ≥2000 ng/mL group, where 68.2% carried at least two underlying diseases and the mean climbed to 2.2 ± 1.1 (Table 9).

**Figure 1:**
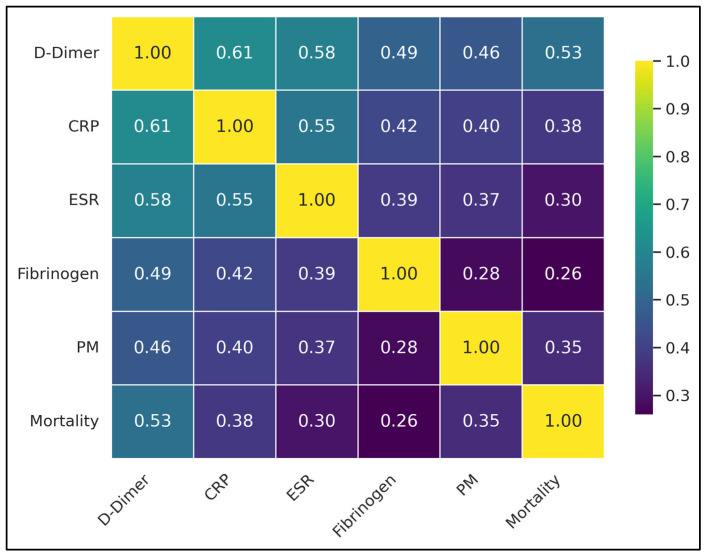
Correlation matrix illustrating the relationships between coagulation and inflammatory markers (D-dimer, CRP, ESR, and fibrinogen), presence of pulmonary microthrombi (PM; binary), and patient mortality (binary outcome). The values indicate Pearson correlation coefficients, with darker colors (purple) representing weaker correlations and brighter colors (yellow) indicating stronger correlations.

Notably, D-dimer showed significant positive correlations with all other variables: CRP (ρ = 0.61), ESR (ρ = 0.58), fibrinogen (ρ = 0.49), pulmonary microthrombi (ρ = 0.46), and mortality (ρ = 0.53), indicating statistical significance. Further, CRP also exhibited significant positive correlations with ESR (ρ = 0.55), fibrinogen (ρ = 0.42), pulmonary microthrombi (ρ = 0.40), and mortality (ρ = 0.38). ESR correlated with fibrinogen (ρ = 0.39), pulmonary microthrombi (ρ = 0.37), and mortality (ρ = 0.30). Fibrinogen showed weaker, yet significant correlations with pulmonary microthrombi (ρ = 0.28) and mortality (ρ = 0.26), but these were not significant. Pulmonary microthrombi and mortality also correlated significantly with a ρ value of 0.35, as presented in Table 10 and Figure 1.

**Figure 2:**
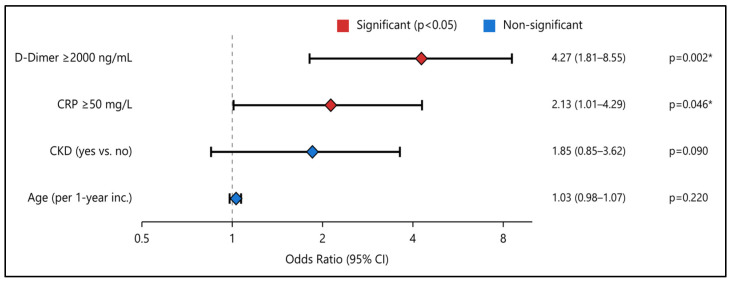
Logistic regression for mortality predictors. Forest plot showing odds ratios (ORs) and 95% confidence intervals (CIs) for clinical variables: chronic kidney disease (CKD), age (per 1-year increment), C-reactive protein (CRP) levels ≥ 50 mg/L, and D-dimer levels ≥ 2000 ng/mL. Vertical dashed line at OR = 1 indicates no effect; values > 1 signify higher odds for the outcome; * indicates statistical significance.

Table 11 summarizes a multivariable logistic regression examining independent predictors of in-hospital mortality. D-dimer ≥ 2000 ng/mL is the strongest indicator, conferring an odds ratio (OR) of 4.27 (95% CI: 1.81–8.55, *p* = 0.002). CRP ≥ 50 mg/L also emerges as independently predictive (OR 2.13, *p* = 0.046), reinforcing the significance of systemic inflammation. By contrast, age shows a modest but non-significant trend (OR 1.03, *p* = 0.22). CKD nearly doubles the odds of mortality (OR 1.85), but the wide confidence interval crosses unity (0.85–3.62), limiting statistical certainty (*p* = 0.09), as presented in Figure 2.

## 4. Discussion

### 4.1. Analysis of Findings

Our study demonstrates strong associations between COVID-19 coagulopathy (as measured via D-dimer) and both multi-organ pathology and adverse clinical outcomes. High D-dimer levels correlated with a greater prevalence of pulmonary microthrombi, bronchopneumonia, and rising inflammatory markers, consistent with prior autopsy data, indicating that severe COVID-19 is characterized by extensive alveolar and endothelial damage [9,10]. Beyond the well-recognized impact of age, we demonstrate that a heavier pre-existing comorbidity load—particularly CKD, diabetes, and CAD—clusters in patients with markedly elevated D-dimer, amplifying their susceptibility to multi-organ pathology and death. By stratifying patients into three D-dimer categories, we observed stepwise increases in organ pathology, ICU admissions, and mortality. These findings bolster the concept that severe hypercoagulability fosters both macro- and microvascular complications, amplifying tissue hypoxia and inflammatory injury [4,15].

In terms of specific organ involvement, the lungs remain the principal site where hypercoagulability and systemic inflammation converge, manifesting as microthrombi, diffuse alveolar damage, and superimposed bronchopneumonia. Patients in the highest D-dimer group displayed substantially higher CRP, reflecting a vicious cycle: local endothelial disruption and microthrombi intensify hypoxia, thereby exacerbating cytokine release and systemic inflammation [5,17]. Cardiac findings such as myocardial fibrosis or recent infarction, as well as renal tubular necrosis, also rose in frequency across escalating D-dimer strata, underscoring how microvascular compromise can escalate to significant organ dysfunction [11].

We also highlight that certain lesions—such as hepatic steatosis—may partly predate COVID-19, but appear exacerbated under acute stress and hypercoagulability. Our correlation matrix and regression models confirm that D-dimer and CRP remain powerful prognostic markers even when adjusted for age and CKD. Clinically, these data advocate for routinely measuring D-dimer early in hospitalization. Patients in the ≥2000 ng/mL group, particularly if accompanied by high CRP, represent a population at imminent risk, meriting aggressive thromboprophylaxis and supportive care. The interplay of coagulopathy and inflammation calls for a multipronged therapeutic approach, potentially pairing anticoagulation with immunomodulatory strategies to attenuate tissue-damaging processes in the lungs, heart, and other vital organs.

In a similar manner, the study by Short et al. [21] found that elevated D-dimer levels were significantly associated with increased mortality in critically ill patients with COVID-19. Conducted across 68 ICUs in the United States, this multicenter cohort study included 3418 critically ill adults, of whom 93.6% had D-dimer concentrations above the normal upper limit. Among these patients, 34.5% died within 28 days. Specifically, patients in the highest D-dimer category (≥8 × the upper limit of normal) had 3.11-fold higher odds of death compared to those in the lowest category in univariate analyses. This association decreased to 1.81-fold increased odds of death after adjustments for demographics, comorbidities, and illness severity were made. Even further adjustment for therapeutic anticoagulation only slightly reduced this relationship to an odds ratio of 1.73. These findings underscored the critical role of hypercoagulability, as indicated by high D-dimer levels, in the mortality risk of severely ill COVID-19 patients [22].

Moreover, the study by Elsoukkary et al. [23] highlighted the severe pulmonary pathology and widespread thromboembolic disease in deceased COVID-19 patients. The autopsy results from 32 individuals showed a high prevalence of diffuse alveolar damage in 94% of cases and extensive thromboembolic disease in 88% of the patients, with notable pathologies also observed in the liver and lymph nodes. The cohort predominantly consisted of older adults, with a mean age of 68, and nearly all had significant comorbidities, averaging four per patient. Similarly, the study by Mikhaleva et al. [24] examined 100 deceased COVID-19 patients and found diffuse alveolar damage to be pathognomonic of COVID-19 viral pneumonia, highlighting it as a primary cause of death, particularly in younger patients. This study also noted widespread intravascular thrombosis and extrapulmonary pathology primarily in the liver and spleen, emphasizing the extensive impact of SARS-CoV-2 beyond the lungs. Both studies underscore the complex interplay of thromboembolism, multi-organ involvement, and severe pulmonary damage as critical factors in COVID-19 mortality, pointing to the need for targeted therapeutic strategies to manage these severe manifestations.

In a similar manner, the study by Maiese et al. [25] reviewed autopsy findings from COVID-19-related deaths, emphasizing the predominant histological features such as diffuse alveolar damage with hyaline membrane formation and microthrombi in small pulmonary vessels. Their literature review, encompassing 28 scientific papers and a total of 341 cases, underscored the high incidence of deep vein thrombosis and pulmonary embolism, suggesting significant endothelial involvement in COVID-19 fatalities. This finding highlights the critical role of endothelial damage in the pathophysiology of COVID-19, reflecting a systemic impact beyond the respiratory system. Correspondingly, the 3D study by Varikasuvu et al. [26], which included a systematic review and meta-analysis of 100 studies involving 38,310 patients, found a significant correlation between elevated D-dimer levels and the risk of disease progression and death in COVID-19 patients. Their analysis revealed that patients with higher initial D-dimer levels had substantial risk of disease progression, with an unadjusted odds ratio of 3.15 and an adjusted odds ratio of 1.64 for overall disease progression. Both studies, through different methodological approaches, highlight the importance of coagulation markers like D-dimer as prognostic tools and underscore the need for integrating these findings into clinical management strategies to mitigate the severe outcomes of COVID-19. Nevertheless, the interpretation of this study’s results should account for multiple comorbid conditions and patient risk factors that can alter these findings [27,28,29,30,31].

### 4.2. Study Limitations

Several limitations warrant consideration. First, although our dataset incorporates rich pathological data, the overall sample size (*n* = 69) may limit the generalizability and statistical power of certain subgroup comparisons. Second, the retrospective nature of data collection precludes the standardization of treatment regimens; variation in anticoagulation or antiviral approaches could have influenced both laboratory values and outcomes. Third, the partial reliance on autopsy data might introduce selection bias: those who underwent post-mortem examination could differ systematically from surviving or non-autopsied patients. Fourth, pathology findings such as myocardial fibrosis or hepatic steatosis can be chronic processes, not exclusively related to COVID-19, and although acute on chronic injury, they cannot be excluded. Fifth, D-dimer was measured at admission for most patients; dynamic changes over time remain unaccounted for. Because only cases with available pathology were eligible, a spectrum severity bias cannot be ruled out; autopsied decedents may overrepresent fulminant disease. Prospective and multicenter research is needed to confirm these associations and examine whether targeted therapies for hypercoagulability reduce pathological organ damage and improve survival. Finally, pathologies such as myocardial fibrosis and hepatic steatosis may reflect pre-existing chronic disease; without pre-infection imaging or biopsy, definitive attribution to SARS-CoV-2 is impossible. However, a notable strength is that biomarker sampling preceded hospital-related immobilization, mitigating the confounding elevation seen after prolonged bed rest.

## 5. Conclusions

In this retrospective cohort, it was found that marked elevations in D-dimer (≥2000 ng/mL) align with more extensive pathological changes in the lungs, heart, kidneys, and liver. Pulmonary microthrombi and bronchopneumonia, in particular, surged in frequency as D-dimer levels rose, potentially reflecting a synergistic interplay between hypercoagulability and systemic inflammation. Concordantly, higher CRP, ESR, and fibrinogen levels paralleled the escalation of D-dimer, forming a composite picture of severe immune-thrombotic dysregulation. Clinically, these pathological findings translated into significantly higher ICU admission rates, prolonged hospital stays, and an increased risk of mortality in patients with levels ≥ 2000 ng/mL. Accordingly, we propose that patients presenting with D-dimer ≥ 2000 ng/mL receive prompt, enhanced thromboprophylaxis and closer organ function surveillance from the first day of admission.

## Figures and Tables

**Table 1:** Demographics, comorbidities, and summary of major pathological findings.

Variable	Overall (*n* = 69)	Pathological Finding	*n* (%) of 69 Patients
Age, mean ± SD (years)	62.8 ± 13.9	Pulmonary microthrombi	26 (37.7)
Male sex, *n* (%)	41 (59.4)	Diffuse alveolar damage	30 (43.5)
Hypertension, *n* (%)	36 (52.2)	Bronchopneumonia	28 (40.6)
Diabetes mellitus, *n* (%)	15 (21.7)	Myocardial fibrosis	31 (44.9)
Chronic kidney disease, *n* (%)	14 (20.3)	Recent myocardial infarction	11 (15.9)
Any malignancy, *n* (%)	12 (17.4)	Renal tubular necrosis	23 (33.3)
-	-	Hepatic steatosis	19 (27.5)

Abbreviations: SD, standard deviation; *n*, number of patients. Percentages are expressed as *n* (%) of the total study cohort.

**Table 2:** Baseline comorbidities stratified by D-dimer category.

Comorbidity	D-Dimer < 500 ng/mL (*n* = 20)	D-Dimer 500–2000 ng/mL (*n* = 27)	D-Dimer ≥ 2000 ng/mL (*n* = 22)	*p*-Value
Hypertension	9 (45.0%)	15 (55.6%)	12 (54.5%)	0.64
Diabetes mellitus	2 (10.0%)	6 (22.2%)	7 (31.8%)	0.14
Chronic kidney disease	1 (5.0%)	5 (18.5%)	8 (36.4%)	0.01 *
Coronary-artery disease	2 (10.0%)	4 (14.8%)	6 (27.3%)	0.24
Obesity (BMI ≥ 30 kg/m^2^)	3 (15.0%)	5 (18.5%)	7 (31.8%)	0.31
COPD	1 (5.0%)	3 (11.1%)	4 (18.2%)	0.28
Active malignancy	3 (15.0%)	5 (18.5%)	4 (18.2%)	0.91
Mean no. of comorbidities ± SD	1.3 ± 0.9	1.7 ± 1.0	2.2 ± 1.1	0.006 *

Abbreviations: BMI, body mass index; CKD, chronic kidney disease; COPD, chronic obstructive pulmonary disease; SD, standard deviation; and *n*, number of patients. *p*-values derive from χ^2^ tests for categorical variables and one-way ANOVA for the mean number of comorbidities. An asterisk (*) denotes statistical significance at *p* < 0.05. Because patients frequently had ≥1 comorbidity, column totals exceed 100%.

**Table 3:** D-dimer group distribution.

D-Dimer Group	Definition (ng/mL)	Patients, *n* (%)
Group 1: Low/normal	<500	20 (29.0)
Group 2: Moderately elevated	500–2000	27 (39.1)
Group 3: Markedly elevated	≥2000	22 (31.9)

Abbreviations: D-dimer, fibrin degradation product measured in nanograms per milliliter (ng/mL); *n*, number of patients. Group definitions follow previously validated COVID-19 risk stratification cut-offs.

**Table 4:** Organ pathology frequency across D-dimer groups.

Pathology	Group 1: <500 (*n* = 20)	Group 2: 500–2000 (*n* = 27)	Group 3: ≥2000 (*n* = 22)	*p*-Value
Pulmonary microthrombi (%)	20.0 (4/20)	37.0 (10/27)	54.5 (12/22)	0.02 *
Bronchopneumonia (%)	25.0 (5/20)	37.0 (10/27)	59.1 (13/22)	0.04 *
Myocardial fibrosis (%)	30.0 (6/20)	48.1 (13/27)	54.5 (12/22)	0.09
Renal tubular necrosis (%)	20.0 (4/20)	33.3 (9/27)	45.5 (10/22)	0.08
Hepatic steatosis (%)	15.0 (3/20)	29.6 (8/27)	36.4 (8/22)	0.13

Abbreviations: D-dimer, fibrin degradation product; *n*, number of patients. Percentages are calculated within each D-dimer stratum. *p*-values are from χ^2^ tests comparing across the three strata. * *p* < 0.05 indicates statistical significance. Organ pathologies are not mutually exclusive.

**Table 5:** Inflammatory markers by D-dimer group.

Marker	Group 1: <500 (*n* = 20)	Group 2: 500–2000 (*n* = 27)	Group 3: ≥2000 (*n* = 22)	*p*-Value
CRP (mg/L), median (IQR)	24.5 (12.3–38.1)	56.2 (28.9–79.0)	92.4 (48.6–110.7)	<0.001 *
ESR (mm/h), median (IQR)	25 (12–40)	40 (20–63)	58 (36–85)	0.002 *
Fibrinogen (mg/dL), mean ± SD	348 ± 95	422 ± 88	478 ± 107	0.001 *

Abbreviations: CRP, C-reactive protein; ESR, erythrocyte sedimentation rate; IQR, inter-quartile range; SD, standard deviation; and *n*, number of patients. CRP and ESR are shown as medians (IQR) owing to non-normal distributions; fibrinogen is presented as mean ± SD. *p*-values come from Kruskal–Wallis tests for non-parametric data and one-way ANOVA for normally distributed variables. * *p* < 0.05 denotes significance after Bonferroni correction.

**Table 6:** Comparative clinical outcomes by D-dimer group.

Outcome	Group 1: <500 (*n* = 20)	Group 2: 500–2000 (*n* = 27)	Group 3: ≥2000 (*n* = 22)	*p*-Value
ICU admission, *n* (%)	4 (20.0)	9 (33.3)	12 (54.5)	0.01 *
Mechanical ventilation, *n* (%)	2 (10.0)	6 (22.2)	8 (36.4)	0.06
Mortality, *n* (%)	1 (5.0)	4 (14.8)	8 (36.4)	0.01 *
Median length of stay (days)	9 (7–12)	13 (10–15)	15 (13–19)	0.004 *

Abbreviations: ICU, intensive care unit; LOS, length of hospital stay; and *n*, number of patients. χ^2^ tests were used for categorical outcomes and Kruskal–Wallis tests for LOS. * *p* < 0.05 indicates statistical significance.

**Table 7:** Subgroup analysis by chronic kidney disease (CKD) status: organ pathologies and outcomes.

Variable	CKD Present (*n* = 14)	CKD Absent (*n* = 55)	*p*-Value *
Pulmonary microthrombi, *n* (%)	9 (64.3)	17 (30.9)	0.01
Bronchopneumonia, *n* (%)	10 (71.4)	18 (32.7)	0.003
Myocardial fibrosis, *n* (%)	10 (71.4)	21 (38.2)	0.02
Renal tubular necrosis, *n* (%)	11 (78.6)	12 (21.8)	<0.001
Markedly elevated D-fimer (≥2000), *n* (%)	9 (64.3)	13 (23.6)	0.002
Mortality, *n* (%)	8 (57.1)	5 (9.1)	<0.001

Abbreviations: CKD, chronic kidney disease; *n*, number of patients. All comparisons employ χ^2^ or Fisher’s exact tests, as appropriate. An asterisk (*) flags *p* < 0.05 after Holm–Bonferroni adjustment for multiple testing. Because patients frequently had ≥1 comorbidity, column totals exceed 100%.

**Table 8:** Subgroup analysis by diabetes mellitus status.

Variable	Diabetes Present (*n* = 15)	Diabetes Absent (*n* = 54)	*p*-Value *
Markedly elevated D-dimer (≥2000 ng/mL), *n* (%)	10 (66.7)	12 (22.2)	0.001
Pulmonary microthrombi, *n* (%)	11 (73.3)	15 (27.8)	0.002
Renal tubular necrosis, *n* (%)	9 (60.0)	14 (25.9)	0.02
Myocardial fibrosis, *n* (%)	10 (66.7)	21 (38.9)	0.06
≥2 Organ pathologies, *n* (%)	11 (73.3)	21 (38.9)	0.01
In-hospital mortality, *n* (%)	7 (46.7)	5 (9.3)	<0.001

Abbreviations: *n*, number of patients. Markedly elevated D-dimer is defined as ≥2000 ng/mL. χ^2^ or Fisher’s exact tests were used to calculate *p*-values; * *p* < 0.05 denotes statistical significance following Holm–Bonferroni correction.

**Table 9:** Comorbidity clustering within D-dimer strata.

D-Dimer Group	Patients with 0–1 Comorbidity *n* (%)	≥2 Comorbidities *n* (%)	Mean No. of Comorbidities ± SD
<500 ng/mL	14 (70.0)	6 (30.0)	1.3 ± 0.9
500–2000 ng/mL	11 (40.7)	16 (59.3)	1.7 ± 1.0
≥2000 ng/mL	7 (31.8)	15 (68.2)	2.2 ± 1.1

**Table 10:** Correlation matrix (Spearman’s ρ) among key variables.

Variable	D-Dimer	CRP	ESR	Fibrinogen	Pulm. Microthrombi (Binary)	Mortality (Binary)
D-dimer	1	0.61 *	0.58 *	0.49 *	0.46 *	0.53 *
CRP	0.61 *	1	0.55 *	0.42 *	0.40 *	0.38 *
ESR	0.58 *	0.55 *	1	0.39 *	0.37 *	0.30 *
Fibrinogen	0.49 *	0.42 *	0.39 *	1	0.28	0.26
Pulm. microthrombi (Binary)	0.46 *	0.40 *	0.37 *	0.28	1	0.35 *
Mortality (Binary)	0.53 *	0.38 *	0.30 *	0.26	0.35 *	1

Abbreviations: CRP, C-reactive protein; ESR, erythrocyte sedimentation rate; and ρ, Spearman’s rank-order correlation coefficient. Binary variables are coded 0 = absent, 1 = present. An asterisk (*) indicates correlations significant at *p* < 0.05 (two-tailed).

**Table 11:** Logistic regression for mortality predictors.

Variable	OR (95% CI)	*p*-Value
D-dimer ≥ 2000 ng/mL	4.27 (1.81–8.55)	0.002 *
CRP ≥ 50 mg/L	2.13 (1.01–4.29)	0.046 *
Age (per 1-year inc.)	1.03 (0.98–1.07)	0.22
CKD (yes vs. no)	1.85 (0.85–3.62)	0.09

Abbreviations: OR, odds ratio; CI, confidence interval; CKD, chronic kidney disease; and CRP, C-reactive protein. Multivariable logistic regression was adjusted for age and comorbidities. * *p* < 0.05 signifies independent predictors of in-hospital mortality. Regression was adjusted for age, sex, obesity, and CKD.

## Data Availability

The data presented in this study are available on request from the corresponding author.
